# Clusterin: structure, function and roles in disease

**DOI:** 10.7150/ijms.107159

**Published:** 2025-01-21

**Authors:** Xing Du, Zhongyao Chen, Wei Shui

**Affiliations:** 1 Department of Orthopaedic Surgery, The First Affiliated Hospital of Chongqing Medical University, Chongqing 400016, China.; 2 Chongqing Municipal Health Commission Key Laboratory of Musculoskeletal Regeneration and Translational Medicine, Chongqing 400016, China.; 3 Orthopedic Laboratory of Chongqing Medical University, Chongqing 400016, China.

**Keywords:** clusterin, secreted protein, neurological disorders, fibrosis, metabolism disease

## Abstract

Clusterin (CLU) is a glycoprotein that exists in various forms in cells, including nuclear, cytoplasmic, and secreted types. The relative molecular weight of CLU varies significantly due to differences in glycosylation and cleavage. Although CLU is commonly present in mammalian tissues and body fluids, its expression levels differ markedly under physiological and pathological conditions. The existence forms and molecular sizes of CLU in cells vary greatly, contributing to its diverse functions. For example, CLU can participate in the occurrence and development of neurological, fibrotic, and metabolic diseases by regulating cell endocytosis, apoptosis, and other processes. This article will review the structural characteristics, basic functions, and potential regulatory mechanisms of CLU protein in physiological and pathological processes.

## 1. Introduction

Clusterin (CLU) is a highly glycosylated heterodimeric protein that is widely distributed in human plasma and tissue fluids. Due to the diverse forms of CLU protein in cells, its expression levels vary greatly under physiological and pathological conditions, resulting in different functions in different tissues, organs, and physiological and pathological states. Although there have been several published reviews on this topic, most of them only discussed the function of CLU in specific diseases, such as tumors 1-3, neurodegenerative diseases 3,4, and musculoskeletal diseases 5, without fully exploring the role of CLU in other diseases. Moreover, recent studies have reported that CLU also plays an important role in fibrotic diseases 6,7, metabolic diseases 8, and cardiovascular diseases 9. Therefore, we conducted this review to summarize the structural characteristics, basic functions, and potential regulatory mechanisms of CLU protein in physiological and pathological processes, providing a theoretical basis for the treatment of CLU protein-related diseases.

## 2. The structural characteristics and basic functions of CLU protein

CLU, also known as apolipoprotein J, is ubiquitously expressed in various body tissues and fluids 10. The human CLU gene is located on the short arm of chromosome 8, spanning from region 2 to region 1, covering approximately 16 kb and comprising 11 exons. Under normal conditions, the full-length mRNA encoding CLU initiates translation from the start codon in exon 2, producing a polypeptide chain of 449 amino acids. This nascent polypeptide is guided into the endoplasmic reticulum (ER) by an N-terminal signal peptide, where it undergoes N-glycosylation and other post-translational modifications, resulting in a glycosylated CLU protein with a molecular weight of approximately 60 kDa. Subsequently, CLU is transported to the Golgi apparatus for further processing, including peptide cleavage and additional glycosylation, before being secreted into the extracellular space via secretory vesicles 11. The secreted CLU protein has a molecular size ranging from 70 to 80 kDa. However, due to varying degrees of glycosylation, SDS-PAGE gel electrophoresis reveals two distinct bands: one corresponding to the intact, uncleaved CLU at approximately 60 kDa, and the other representing the cleaved α and β chains at approximately 40 kDa 12. CLU is a highly glycosylated protein, with carbohydrates accounting for about 30% of its total mass. The N-linked glycans of CLU primarily consist of a core of three mannose residues and are extensively modified with fucose. These glycans play crucial roles in cellular communication and immune function regulation 13.

**Figure 1:**
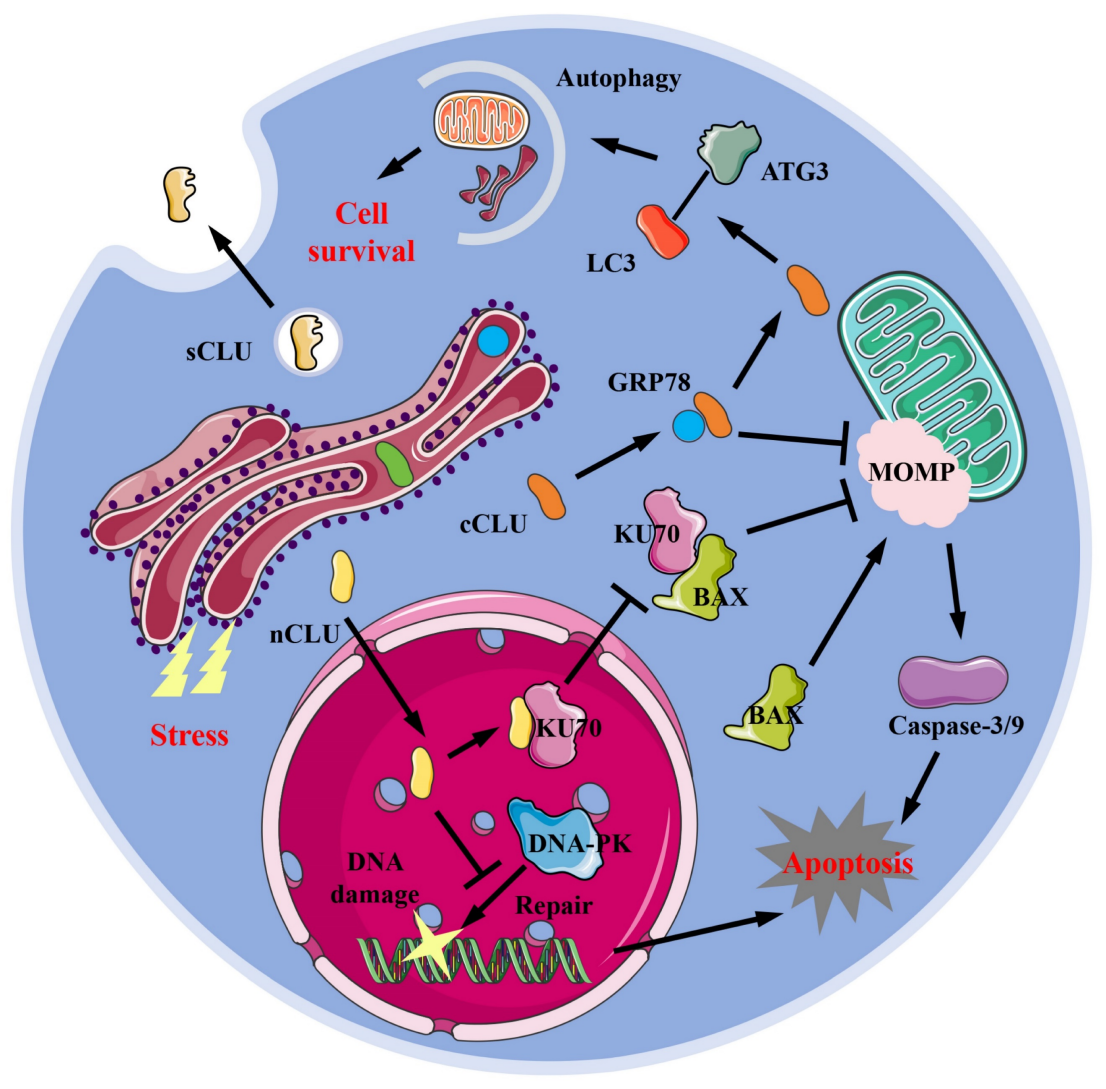
** Forms and potential mechanisms in physiological functions of CLU protein.** sCLU, secreted type clusterin; cCLU, cytoplasmic type clusterin; nCLU, nucleus type clusterin; LC3, microtubule-associated protein 1 light chain 3; ATG3, autophagy-related protein 3; GRP78, glucose regulated protein 78; MOMP, mitochondrial outer membrane perforation; KU70, X-ray repair cross complementing 6; BAX, BCL2 associated X; DNA-PK, DNA-dependent protein kinase.

CLU exists in various forms within cells, including the secreted type (sCLU), the cytoplasmic type (cCLU), and the nucleus type (nCLU). sCLU is a highly glycosylated protein, primarily found in blood and cerebrospinal fluid, and is also distributed in other tissues. Under physiological conditions, the concentration of sCLU protein in plasma ranges from 35-105 μg/mL 14, while in cerebrospinal fluid, it is between 1.2-3.6 μg/mL 15. Due to hydrophobic regions, sCLU typically exists stably in body fluids as dimers or tetramers. sCLU participates in normal physiological processes such as complement regulation 16 and lipid transport 17. Under pathological conditions, such as Alzheimer's disease (AD) 18-20, fibrosis 6,7, cancer 21,22, and cardiovascular disease 8,23, the local concentration of sCLU protein often increases. Under normal physiological conditions, cells generally produce sCLU protein, but when stimulated by the external environment, some of the produced CLU protein remains within the cell. This form of CLU is known as intracellular CLU (iCLU). The iCLU protein has two physiological structures within the cell: one consists of two chains with lower glycosylation levels than sCLU, known as cCLU; the other type is that the formed peptide chains do not undergo cleavage and N-glycosylation modification, and typically function within the nucleus 22, known as nCLU. The common function of cCLU protein is to inhibit cell apoptosis. Research has shown that drugs such as paclitaxel or MG132 treatment can induce stress responses in the endoplasmic reticulum of cells; the GRP78 (glucose regulated protein 78) protein in the endoplasmic reticulum binds to cCLU, stabilizing its conformation and transporting it to the periphery or interior of mitochondria 24. The cCLU transported to the periphery of mitochondria can inhibit mitochondrial outer membrane perforation (MOMP), thereby preventing the release of cytochrome C and inhibiting cell apoptosis 24,25 **(Figure 1)**. Additionally, cCLU can bind to the LC3 (microtubule-associated protein 1 light chain 3)-ATG3 (autophagy-related protein 3) complex, promoting the esterification of LC3 protein, thereby enhancing cell autophagy ability 26 and resisting cell apoptosis. Conversely, nCLUs, which also belong to the same iCLU, promote cell apoptosis. For instance, nCLU directly binds to Ku70 (X-ray repair cross complementing 6) protein, reducing the complex formed by Ku70 and BAX (BCL2 associated X), causing free BAX to induce intracellular mitochondrial outer membrane perforation, thereby activating downstream apoptotic pathways 27; nCLU can also inhibit the repair of X-rays induced DNA damage by binding to DNA-PK (DNA dependent kinase complexes), thereby activating downstream apoptosis processes in cancer cells 28 **(Figure 1)**. Therefore, classifying and studying the different existence forms of CLU helps to understand the complex mechanisms of CLU and its role in different diseases, which is of great benefit in elucidating the contradictory effects of CLU shown in previous studies.

## 3. The role of CLU protein in neurological diseases

### 3.1 Alzheimer's disease

**Figure 2:**
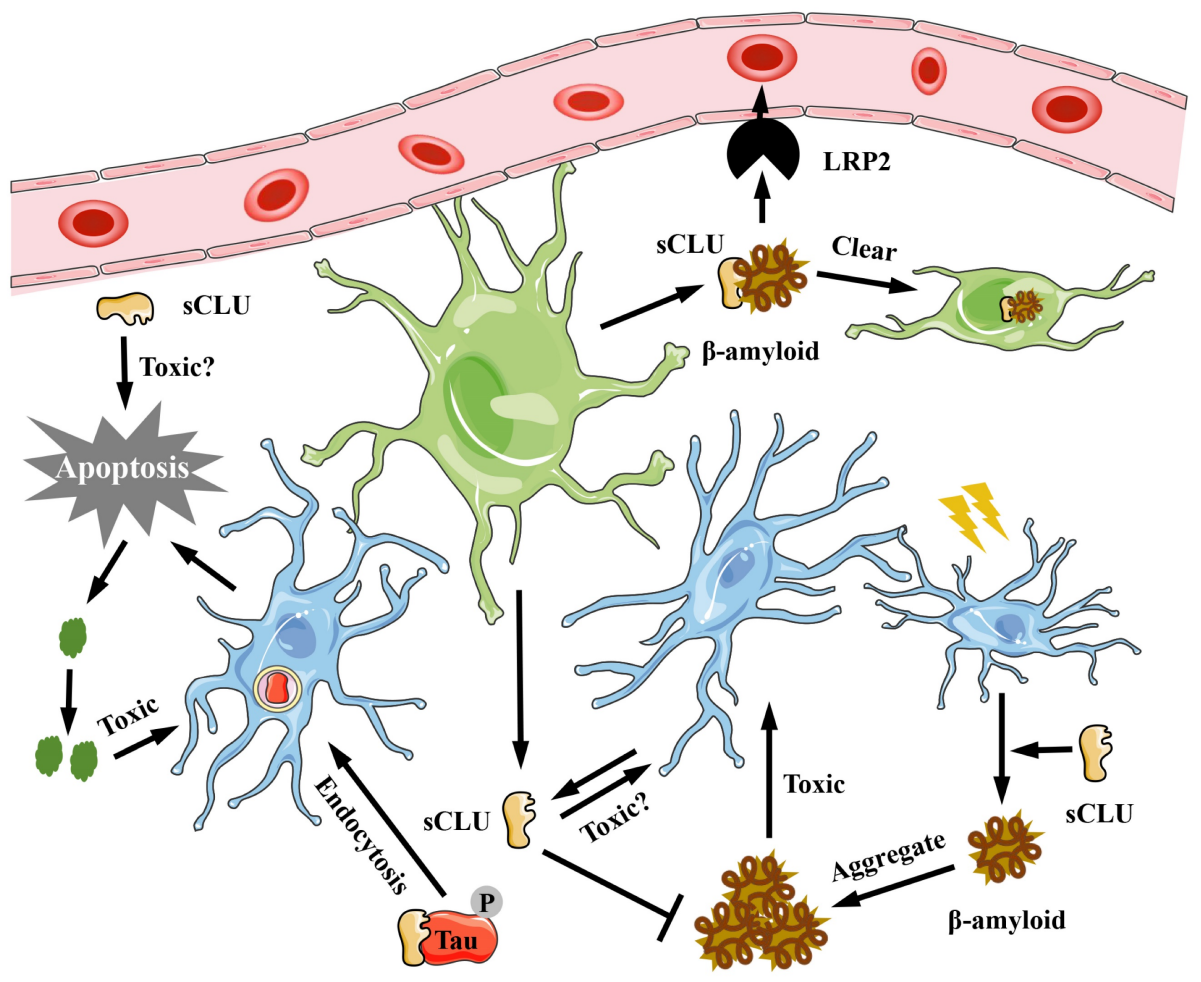
** The role of CLU protein in Alzheimer's disease.** In neural tissue, sCLU is secreted by neural cells and glial cells. The sCLU can inhibit β-amyloid protein aggregation, promote its transport to microglia and being cleared, and thus reduce its toxicity. Moreover, sCLU can also bind to phosphorylated Tau to exert its toxic effects on nerve cells. sCLU, secreted type clusterin; LRP2, lipoprotein receptor-related protein 2.

The CLU protein, primarily expressed and secreted by neuronal cells and astrocytes, is found in cerebrospinal fluid (CSF). Research indicates that the concentration of CLU protein in the CSF of Alzheimer's disease (AD) patients is approximately 40% higher than in normal individuals 29. Furthermore, the plasma concentration of CLU protein is inversely related to the cognitive ability of AD patients 30 but directly proportional to the incidence and severity of AD 31. A single nucleotide polymorphism (SNP) in high-risk genes associated with AD has shown a strong correlation between the rs11136000 mutation in the CLU gene and the delayed onset of AD. Immunofluorescence staining of brain tissue from AD patients reveals that CLU protein co-localizes with β-amyloid protein 12,19,29, suggesting an interaction between the two. Additionally, direct injection of recombinant CLU protein into the cerebrospinal fluid of AD model mice, using amyloid precursor protein (APP) mutant mice, can reduce the deposition of amyloid protein in the brain 32. Research has found that the primary mechanism by which CLU protein alleviates Alzheimer's disease may involve binding to low-density lipoprotein receptor-related protein 2 (LRP2), triggering receptor expressed on myeloid cells 2 (TREM2), or heparan sulfate (HS) on the membrane surface, thereby mediating the clearance of β-amyloid and its polymers in the extracellular matrix 19,33,34 **(Figure 2)**. Moreover, studies have shown that the concentration of CLU protein in the plasma of mice increases significantly after exercise, which can inhibit neuronal apoptosis and hippocampal inflammation, thereby improving cognitive ability in mice 35. However, in recent years, studies have suggested that CLU protein may promote the progression of AD 36. Excessive phosphorylation of Tau protein is another typical feature of AD. Research has demonstrated that CLU protein can bind to overly phosphorylated Tau protein, inhibiting Tau protein hydrolysis in lysosomes, leading to Tau aggregation, and causing cell rupture and death 36 **(Figure 2)**. Additionally, after the membrane of dead cells ruptures, CLU-Tau is released and aggregates outside the cell, forming pathological plaques 36 **(Figure 2)**. In the brain tissue of Clu-knockout mice with cerebral ischemia, Han et al. 37 found that the area of hypoxic stress damage was smaller than in control mice, indicating that CLU protein is not beneficial for neuronal survival under stress. The latest study on AD also suggests that CLU is an important cause of pathological plaques 38. Therefore, the role of CLU in AD cannot be generalized, as there has not been a comprehensive localization and expression analysis of CLU in intracellular studies thus far. Consequently, analyzing its function solely from the perspective of protein expression and disease progression is relatively narrow and incomplete. Although Herring et al. 39 analyzed the expression positions and levels of different RNA types of CLUs in various cells of neural tissue in detail, there remains a lack of comprehensive research in this area.

### 3.2 Parkinson's disease

Parkinson's disease (PD) is a highly prevalent degenerative disease of the central nervous system, primarily caused by the formation and accumulation of Lewy bodies in dopamine neurons within the substantia nigra of the midbrain, leading to extensive neuronal death. Research has found that the *Clu* gene rs11136000 is associated with disease development in PD patients 40. Analyzing the exosome components of nerve cells in the blood of PD patients, it was discovered that the decrease of CLU content in the blood was closely related to the occurrence of PD 41. The aggregation of α-synuclein proteins is a crucial step in the formation of Lewy bodies. Immunofluorescence staining results showed that in the brain tissue of PD patients, the CLU protein was mainly distributed at the α-synuclein site of the Lewy body 42. The main mechanism is that the cCLU protein can interact and bind with the hydrophobic regions on the surface of α-synuclein proteins, weakening their aggregation ability, thereby reducing the formation of Lewy bodies and ultimately decreasing neuronal apoptosis 43. On the other hand, during the clearance of extracellular α-synuclein proteins and their oligomers by astrocytes, extracellular sCLU would bind to α-synuclein proteins and inhibit the phagocytosis of astrocytes, leading to an increased concentration of extracellular α-synuclein proteins 44, which may, in turn, exacerbate the development of PD.

## 4. The role of CLU protein in fibrosis related diseases

**Figure 3:**
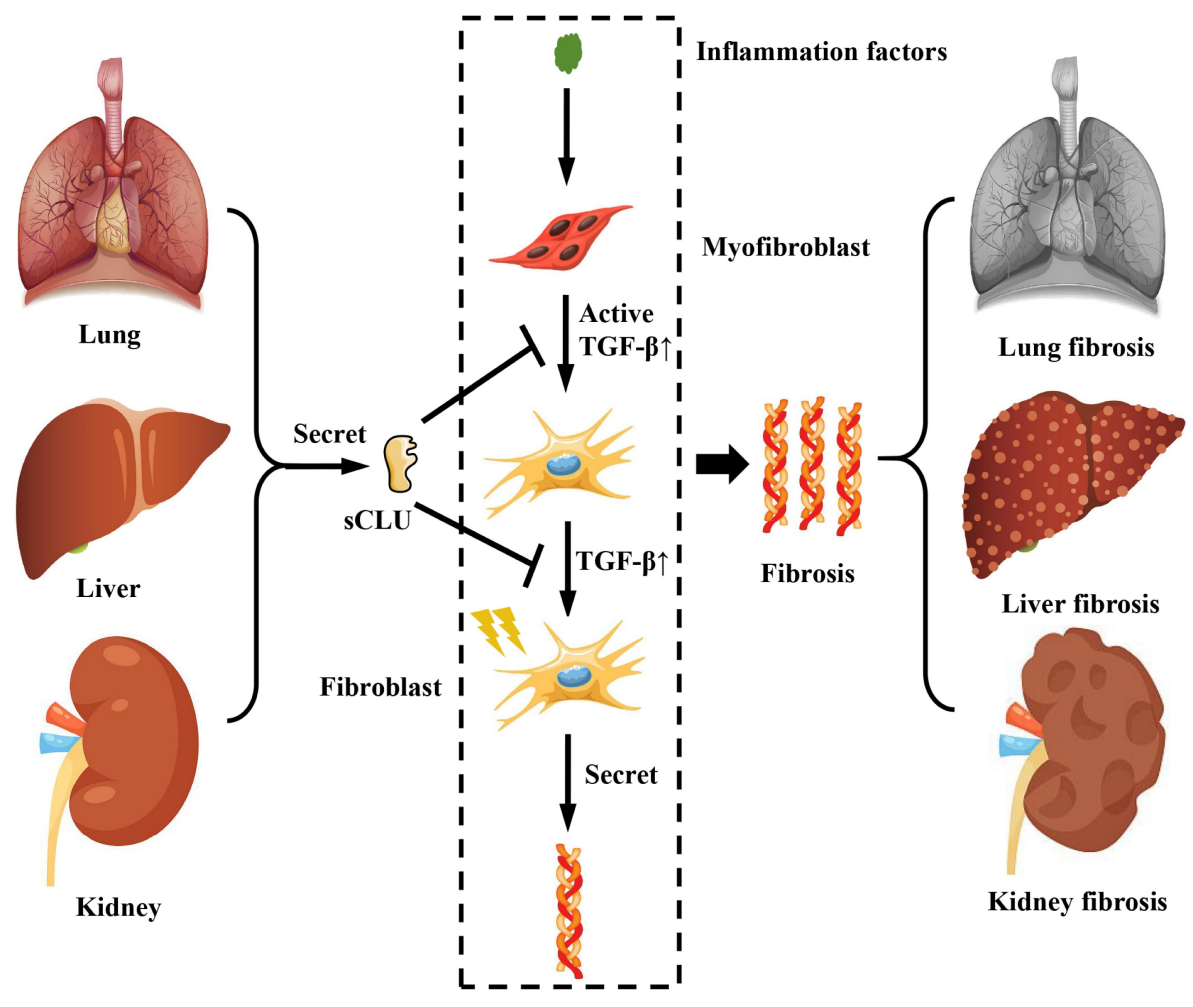
** The role of CLU protein in fibrosis related diseases.** The sCLU secreted by the lung, kidney and liver can inhibit the activation of fibroblasts and the generation of collagen fibers by suppressing the TGF-β pathway. sCLU, secreted type clusterin; TGF-β, transforming growth factor β.

The hallmark of fibrosis is pathological changes resulting from the excessive deposition of extracellular matrix components, such as collagen. In a mouse model of renal fibrosis induced by unilateral ureteral obstruction (UUO), the expression of CLU in the kidney was observed to increase during the progression of renal fibrosis, and a corresponding rise in CLU concentration in urine was detected 45. Furthermore, the creation of a UUO model in Clu gene knockout mice revealed a more rapid progression of fibrosis 45. Conversely, in a mouse model of UUO renal fibrosis with *Clu* overexpression, it was discovered that the overexpression of *Clu* could suppress the development of renal fibrosis 46. Studies on pulmonary fibrosis have also demonstrated that the CLU protein can inhibit the progression of pulmonary fibrosis 6, primarily by reducing the transforming growth factor β (TGF-β) expression, preventing the activation of astrocytes and their differentiation into fibroblasts, as well as the expression and deposition of extracellular matrix proteins like collagen **(Figure 3)**. Clinical studies on patients with acute chronic liver failure (ACLF) caused by HBV have indicated that the concentration of CLU protein in the patient's serum can serve as a marker for disease progression in HBV-ACLF. As the disease advances, the concentration of CLU protein in the patient's serum tends to decrease 47. Research on mouse liver fibrosis found that the expression of CLU protein increased following the onset of liver fibrosis 7; inhibiting the expression of CLU protein was shown to accelerate the progression of liver fibrosis 48. The mechanism involved the inhibition of TGF-β-mediated activation of hepatic stellate cells and the production and deposition of a substantial amount of extracellular matrix. Thus, as an extracellular molecular chaperone, CLU not only directly participates in the deposition process of the extracellular matrix but also regulates corresponding cellular physiological processes, playing a crucial role in maintaining extracellular matrix homeostasis.

## 5. The role of CLU in glucose and lipid metabolism

**Figure 4:**
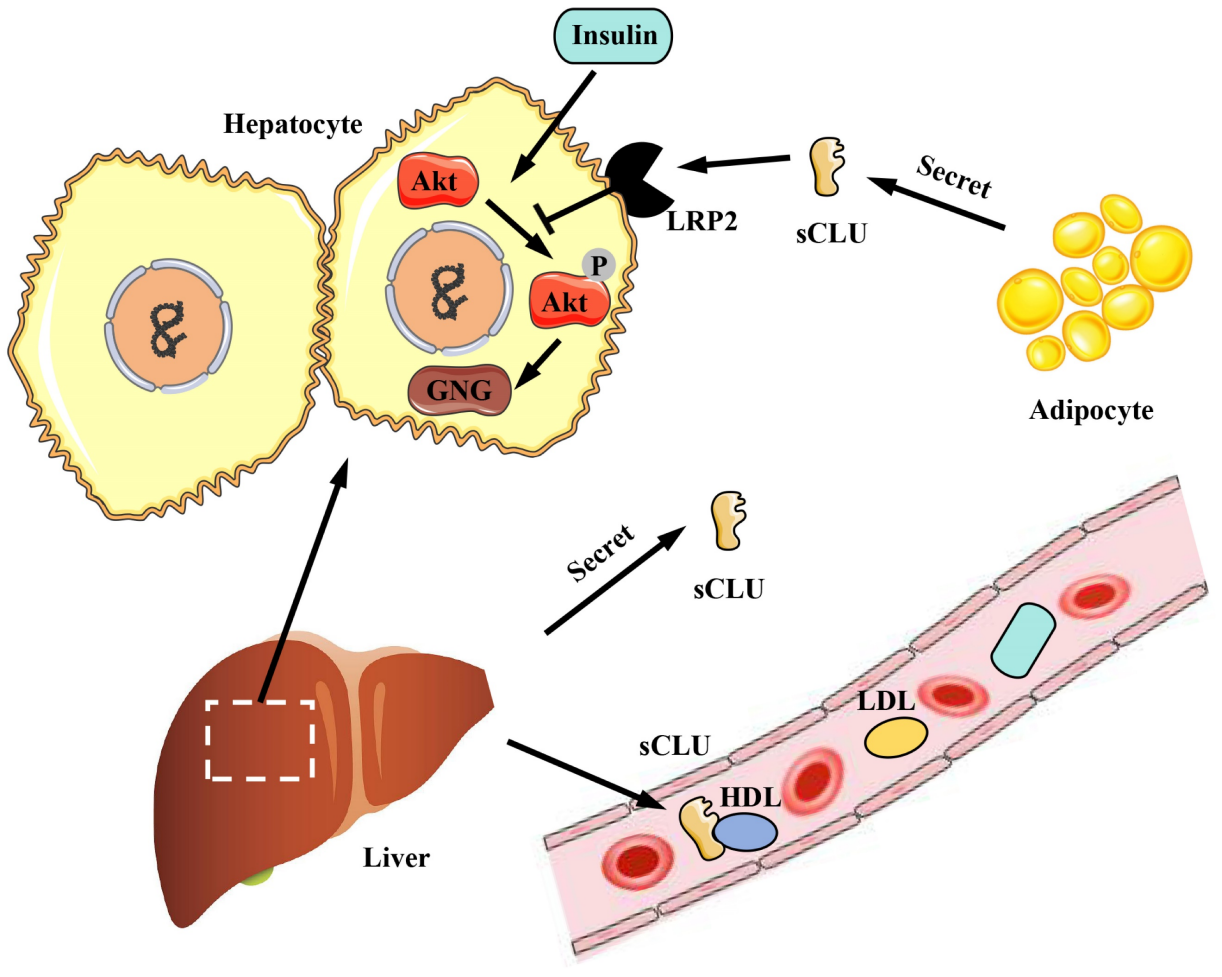
** The role of CLU protein in glucose and lipid metabolism in liver.** The sCLU protein produced by adipocytes can enter hepatocytes through LRP2 and inhibit Akt phosphorylation, thus promoting gluconeogenesis and reducing insulin sensitivity of hepatocytes. Besides, the sCLU secreted by the liver has the similar effect, and can also be transported to the circulatory system to function. sCLU, secreted type clusterin; LRP2, lipoprotein receptor-related protein 2; Akt, protein kinase B; GNG, gluconeogenesis; HDL, high density lipoprotein; LDL, low density lipoprotein.

**Figure 5:**
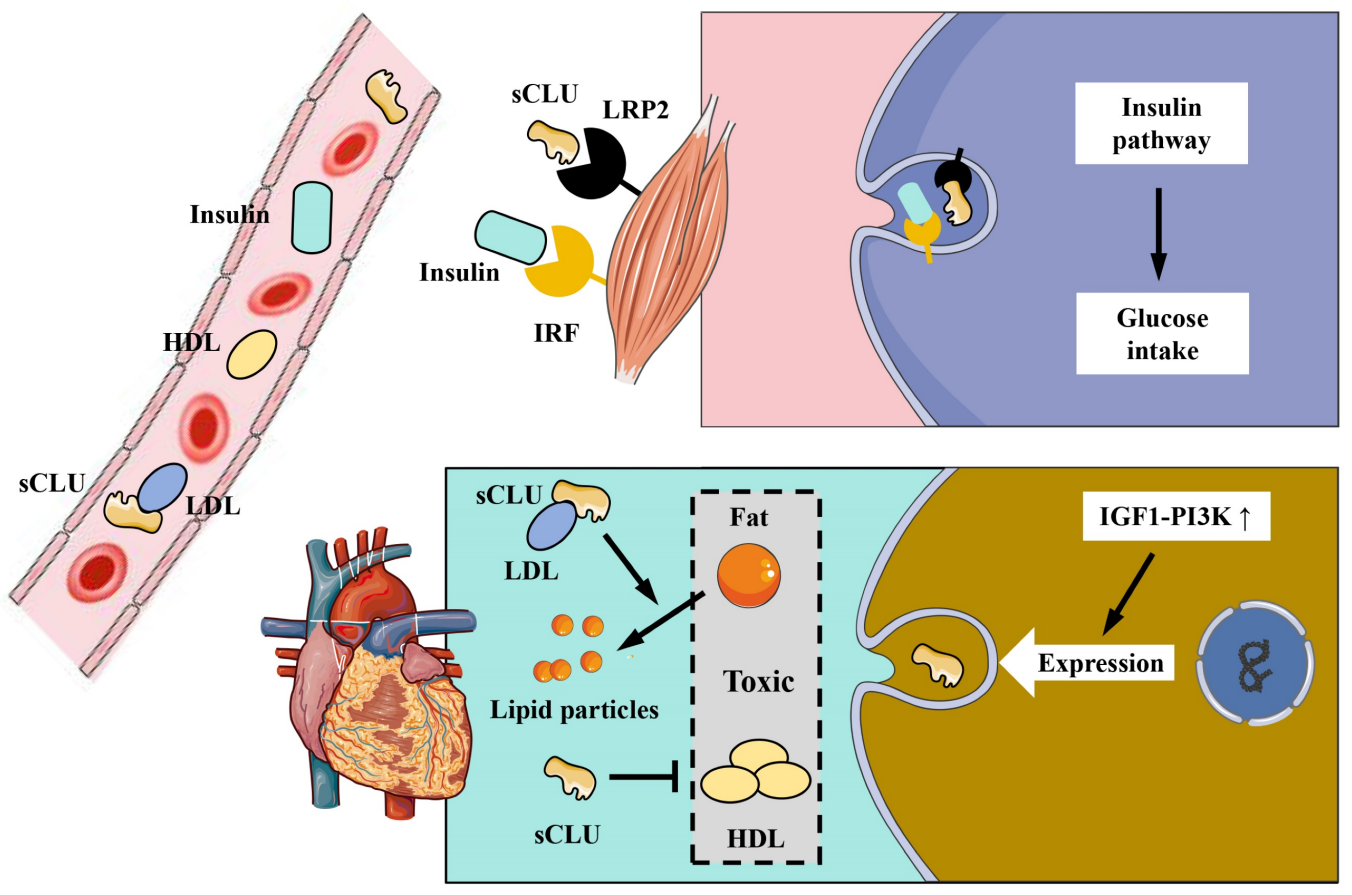
** The role of CLU protein in muscle and the cardiovascular system.** In muscle system, insulin and sCLU can produce synergistic effects to promote the activation of the insulin pathway and enhance the glucose uptake. In cardiovascular system: sCLU can inhibit the aggregation of LDL and reduce its cardiovascular toxicity; HDL and sCLU can promote fat decomposition and then attenuate the cardiovascular damage of fat; activation of the IGF1-PI3K pathway in cardiac cells can promote the generation of sCLU and play a role in protecting cardiomyocytes from the toxicity of fat and LDH. sCLU, secreted type clusterin; LRP2, lipoprotein receptor-related protein 2; IRF, insulin receptor family; HDL, high density lipoprotein; LDL, low density lipoprotein; IGF1, insulin-like growth factor 1; PI3K, phosphatidylinositol 3-kinase.

The CLU protein is primarily expressed and secreted into plasma and tissue fluids by tissues and organs such as the liver and heart. CLU plays a significant role in physiological processes, including metabolism and anti-inflammatory effects. Obesity is associated with a high incidence of diabetes, and diabetic patients often exhibit symptoms related to insulin resistance. Interestingly, the CLU protein has been found to regulate insulin resistance 8. Compared to individuals with normal body weight, obese individuals exhibit increased expression and secretion of the CLU protein in adipocytes 8. The primary mechanism involves the transport of sCLU to the liver, where it binds to the LRP2 receptor on the surface of liver cells, inhibiting insulin-induced Akt phosphorylation and stimulating downstream gluconeogenesis (GNG) pathways. This results in reduced insulin sensitivity of liver cells and impacts the body's metabolism of glucose and lipids 8 **(Figure 4)**. Additionally, as the liver is the main source of CLU in the body, liver-secreted CLU can also interact with the LRP2 receptor on muscle cell membranes, enhancing the activation of downstream pathways by insulin, improving insulin sensitivity in muscle cells, and increasing glucose uptake by muscle tissue 49 **(Figure 5)**. Beyond metabolic regulation, CLU also influences food intake. The CLU protein can act on the LRP2 receptor in the hypothalamus, triggering Stat3 phosphorylation and suppressing central feeding-related pathways 50, leading to weight loss in mice. In summary, CLU is considered a liver regulatory factor and has garnered increasing interest from researchers, offering new insights into the functional interactions between different organs.

## 6. The role of CLU protein in cardio-cerebrovascular diseases

As an apolipoprotein, the CLU protein is an important component of high density lipoprotein (HDL) and plays a crucial role in cardio-cerebrovascular diseases 51. The primary mechanism of HDL's anti-atherosclerotic effect is that the apolipoproteins within HDL particles activate key enzymes of lipoprotein metabolism, promoting liver cells to clear cholesterol from tissues, thereby slowing down and preventing atherosclerosis. The CLU protein is one of these significant apolipoproteins. It has been shown that the CLU protein in HDL from healthy individuals can reduce heart damage by inhibiting the apoptosis of cardiac endothelial cells; however, HDL particles from patient populations lack CLU protein binding 52, suggesting that CLU is involved in the protective effect of HDL on cardiovascular function. The concentration of CLU protein is also related to cardiac injury. Clinical data analysis has found that sCLU in plasma can serve as a marker for myocardial infarction injury, with its concentration decreasing during early ischemic injury of the heart, while the content of iCLU increases 23. Protein components such as CLU in HDL can also indicate the therapeutic effect of stroke. Analysis of HDL protein components in the plasma of stroke patients post-treatment revealed a positive correlation between CLU content and recovery progress 41. Low density lipoprotein (LDL) has the opposite effect of HDL and can promote atherosclerosis. Research has shown that saturated fatty acids can inhibit the binding of CLU protein to LDL, thereby increasing the aggregation of LDL in plasma 53. It has also been reported that the insulin-like growth factor 1-phosphatidylinositol-3-kinase (IGF1-PI3K) pathway has a protective effect on the heart and enhances cardiac function, and the activation of this pathway can increase CLU expression 54 **(Figure 5)**. In a rat myocardial infarction model experiment, the administration of human CLU protein in the blood was shown to reduce the myocardial infarction area by 75% and lower the mortality rate 55. All of the above research results indicate that CLU plays a significant role in the development of cardio-cerebrovascular disease.

## 7. The role of CLU in tumor regulation

Various isoforms of CLU have been reported to have varying effects on cancer cells. Under the stimulation of paclitaxel, the expression of cCLU in prostate cancer cells increased, and cCLU was transported from GRP78 to mitochondria. This process could stabilize the outer membrane of mitochondria and reduce the release of cytochrome C, thereby inhibiting cell apoptosis 24. In osteosarcoma cells, cCLU inhibited the dissociation of Ku70 and BAK complexes and the translocation of BAX to the mitochondrial membrane, stabilizing the outer mitochondrial membrane and preventing MOMP from occurring 56. cCLU can also regulate pathways related to autophagy. In experiments using human prostate cancer cells as models, it was found that cCLU can bind to the LC3-ATG3 complex, promote the lipidation of LC3 protein, and thereby enhance cell autophagy and the ability of cancer cells to resist external environmental stimuli 26. Research has reported that cells with high expression of N-cadherin also had higher levels of cCLU, which was beneficial for the survival of cancer cells 21. In oral cancer cells, overexpression of CLU was beneficial for the activation of the AMPK/Akt/mTOR-guided cell autophagy pathway and improved the survival rate of oral cancer cells 57. However, nCLU exhibited different regulatory functions. Under ionizing radiation, the nCLU produced by cells can bind to Ku70, leading to an increase in intracellular BAK content 27; it can also inhibit the repair of DNA by DNA-PK in damaged cells, thereby enhancing the occurrence of downstream cell apoptosis 22. The TAK1-NF-κB pathway was over-activated in small cell lung cancer patients with *Clu* deletion; in vitro treatment with a TAK1-NF-κB inhibitor can inhibit the generation and accelerate the death of cancer cells 58. CLU also showed the function of inhibiting the aforementioned pathway, which provided more options for diversified treatment of cancer 58.

## 8. The role of CLU in musculoskeletal disorders

Currently, limited research has been conducted on the role of CLU in musculoskeletal diseases. Studies have shown that the expression levels of CLU in the cartilage of osteoarthritis (OA) patients 59, as well as in the serum and synovial fluid of hip OA and knee OA patients 60, were significantly higher compared to those in the normal control population. A recent study further demonstrated that CLU plays a crucial cytoprotective role in OA. Specifically, the knockdown of CLU significantly inhibited chondrocyte proliferation and promoted the expression of markers associated with inflammation and oxidative stress 61. In bone development studies, it was observed that CLU gene expression decreased during the differentiation process of mouse bone marrow mesenchymal stem cells (mBMSCs). sCLU exhibited a dose-dependent inhibitory effect on mBMSC differentiation into osteoblasts and an induction of adipogenic differentiation 62. Additionally, overexpression of CLU was noted in atrophic and degenerated fibers of osteoporotic muscles. Silencing CLU via siRNA restored myoblast proliferation and differentiation capacity, thereby identifying CLU as a marker of muscle degeneration 63. Moreover, increased expression of CLU protein was observed in osteocytes and osteoblasts of osteoporosis (OP) patients, while sCLU levels were reduced 64. sCLU not only hindered BMSC differentiation into osteoblasts by inhibiting the ERK1/2 signaling pathway 62 but also suppressed osteoclastogenesis by reducing the proliferation of M-CSF-dependent osteoclast precursor cells 65. In conclusion, CLU plays a significant role in bone metabolism under various pathological conditions and warrants further in-depth investigation.

## 9. Summary and prospects

Overall, CLU, a protein with potential protective effects on physiological functions, exhibits complex expression patterns and functions across various pathological conditions. Summarizing the expression patterns and signaling pathways of CLU under physiological and pathological conditions can aid in understanding and applying CLU. CLU may serve as a serological marker for neurological diseases, cardio-cerebrovascular diseases, and fibrosis-related diseases, enabling the prediction of disease progression. As an extracellular chaperone protein, CLU's most critical role is to participate in the clearance of extracellular matrix components such as amyloid and collagen, thereby alleviating the occurrence and development of diseases. Although the conclusion that the extracellular CLU protein alleviates neurological disorders like AD remains controversial, a deeper understanding of the mechanism by which CLU functions as a molecular chaperone in extracellular matrix clearance can assist in establishing future prevention and treatment strategies for AD. Additionally, the iCLU protein plays a significant role in the processes of apoptosis and autophagy, potentially alleviating myocardial infarction, diabetes, and other diseases. Consequently, the iCLU and its molecular regulatory mechanisms could serve as potential drug targets for treating these diseases in the future. The function of the CLU protein extends beyond the organs that express it; it is also a protein that interacts with the circulatory system. The CLU protein secreted by the liver can act on muscle tissue and brain neurons, playing a vital regulatory role between organs. The role and mechanism of CLU in tumors are not yet fully understood, and the effects of CLU vary depending on the different forms present in various tumors. Therefore, accurately understanding the impact of CLU on cellular physiology in different environments will help to advance research on tumors and develop new treatment approaches.

## Funding

This work was supported by the China Postdoctoral Science Foundation (2024M763910) and the Natural Science Foundation of Chongqing (CSTB2022NSCQ-MSX0972).

## Abbreviations

CLU: clusterin
ER: endoplasmic reticulum
sCLU: secreted type of clusterin
cCLU: cytoplasmic type of clusterin
nCLU: nucleus type of clusterin
iCLU: intracellular clusterin
AD: Alzheimer's disease
GRP78: glucose regulated protein 78
MOMP: mitochondrial outer membrane perforation
LC3: microtubule-associated protein 1 light chain 3
ATG3: autophagy-related protein 3
Ku70: X-ray repair cross complementing 6
BAX: BCL2 associated X
DNA-PK: DNA dependent kinase complexes
CSF: cerebrospinal fluid
SNP: single nucleotide polymorphism
APP: amyloid precursor protein
LRP2: lipoprotein receptor-related protein 2
TREM2: triggering receptor expressed on myeloid cells 2
HS: heparan sulfate
PD: Parkinson's disease
UUO: unilateral ureteral obstruction
TGF-β: transforming growth factor β
ACLF: acute chronic liver failure
GNG: gluconeogenesis
Akt: protein kinase B
HDL: high density lipoprotein
LDL: low density lipoprotein
IGF1: insulin-like growth factor 1
PI3K: phosphatidylinositol-3-kinase
IRF: insulin receptor family
OA: osteoarthritis
mBMSCs: mouse bone marrow mesenchymal stem cells
OP: osteoporosis
